# The "Dangles" — Wrist, Finger and Thumb Drop: A Case Report of Saturday Night Palsy and a Historical and Molecular Detour

**DOI:** 10.7759/cureus.13195

**Published:** 2021-02-07

**Authors:** Hassan Kesserwani

**Affiliations:** 1 Neurology, Flowers Medical Group, Dothan, USA

**Keywords:** radial nerve injury, medicine history

## Abstract

Saturday night palsy refers to neuropraxia of the radial nerve following prolonged compression against the spiral groove of the humerus. The pattern of weakness is unique with wrist, thumb, and finger drop, and recovery is universal by six months. What makes this clinical entity fascinating are the toxic and metabolic diseases that can manifest similarly, namely, plumbism (Saturnism) and acute porphyrias. The acute porphyrias (heme biosynthetic inborn errors of metabolism) are well known to cause motor neuropathy, with upper more than lower limb weakness, with wrist, thumb and finger drop a frequent manifestation. Intriguingly, lead neurotoxicity (plumbism), which has historically been tightly associated with wrist, thumb, and finger drop, is associated with the inhibition of at least three enzymes of heme biosynthesis. Mechanistically, interference with heme ring synthesis interferes with electron transport chain protein synthesis, which leads to oxidative phosphorylation defects, energy failure, axonal transport impairment, and, subsequently, an axonopathy. The lead atom has a valence of two (giving up two electrons) similar to that of the calcium atom, allowing lead to bind to spongy and cortical bone and interfering with the presynaptic voltage-gated calcium channel (VGCC) neurons. We list the salient features and similarities of these two very rare entities, hence, the term plumboporphyric neuropathy for one of the genetic variants of heme biosynthesis. Lastly, we briefly outline the spectacular history of plumbism and adumbrate on the similarity of the bacchanalian ecstasy of Roman festivals (Saturnism), over-indulgence in lead-sweetened and lead-laden barrels of wine, and the syncretism between the Saturnine palsy and Saturday night palsy. We present a case of wrist, thumb, and finger drop due to compressive neuropraxia as a platform to segue into the historical simulacra.

## Introduction

Saturday night palsy is a compressive neuropathy of the radial nerve against the spiral groove of the humerus leading to neuropraxia, conduction slowing, or conduction block along the compressed segment. It is usually caused by perching of the upper arm against a sharp or blunt object, usually after a night of carousal with alcohol. The prognosis is excellent. The pattern of weakness is typical with wrist, thumb, and finger (WTF) drop. The wrist drop is due to the weakness of the extensor carpi radialis longus (ECRL) while the finger and thumb drop due to the fascicular involvement of the posterior interosseus nerve (PIN). Sensory symptoms over the superficial radial sensory along the radial side of the forearm and thumb may also occur [1-2].

A cervical C7 nerve root impingement can lead to a WTF drop, but this typically implicates the triceps muscle, which is usually spared with a compressive lesion against the spiral groove. A useful clinical pearl is sparing of the ECRL, C6, and C7 nerve roots, and weakness/atrophy of the extensor digitorum communis (EDC), C8, and T1 nerve roots implicating the C8 nerve root (mostly finger drop). This leads us into the differential diagnosis of WTF drop, which can be isolated, as in trauma to the upper arm and local tumors as in lipomas or ganglion cysts. It can also be part of an inflammatory process, as in a mononeuritis multiplex (vasculitis), atypical chronic inflammatory demyelinating polyradiculopathy (CIDP), or multifocal motor neuropathy [3]. An isolated posterior cord plexopathy is very rare. However, these diseases are not the focus of this article. Instead, we will be focusing on the rarer and historically dramatic entities of plumbism (Saturnism) and the acute porphyrias, and the aptly named plumboporphyric neuropathy and its cogeners.

The valence of the lead atom (two electrons available for oxidative reduction) is similar to that of the calcium atom. This provides a platform for the lead atom to interfere with the calcium-dependent processes that are essential for axonal function such as the sodium-potassium adenosine-triphosphatase (Na/K-ATPase) ion pump. Lead also interferes with the synthesis of the heme molecule, which is essential for the formation of hemoglobin, myoglobin, and cytochrome oxidases (electron transport chain in mitochondria). Therein lies the connection between plumbism (lead toxicity) and acute porphyrias (the inborn errors of porphyrin metabolism) [4-5]. It should be noted that both conditions are extremely rare.

We describe a typical case of Saturday night palsy and then segue into the fascinating history of plumbism. In the discussion section, we will address the similarities between the clinical phenotype of plumbism and acute porphyrias and discuss the molecular biology of both entities.

## Case presentation

We present the case of a 53-year-old, healthy, right-handed man who spent an evening drinking beer while watching a game of football. He slept on the bar stool with his right arm overhanging the back of the stool for six hours. He woke up and noted numbness over the radial distal forearm radiating into his thumb, and he was unable to extend his right wrist, thumb, and fingers. The numbness was transient lasting a few hours. He denied neck or arm pain. A visit to the emergency room led to a normal computed tomography (CT) scan of the brain, a prescription for a wrist splint, and a referral to neurology made the following day. His past medical history was unremarkable.

**Figure 1:**
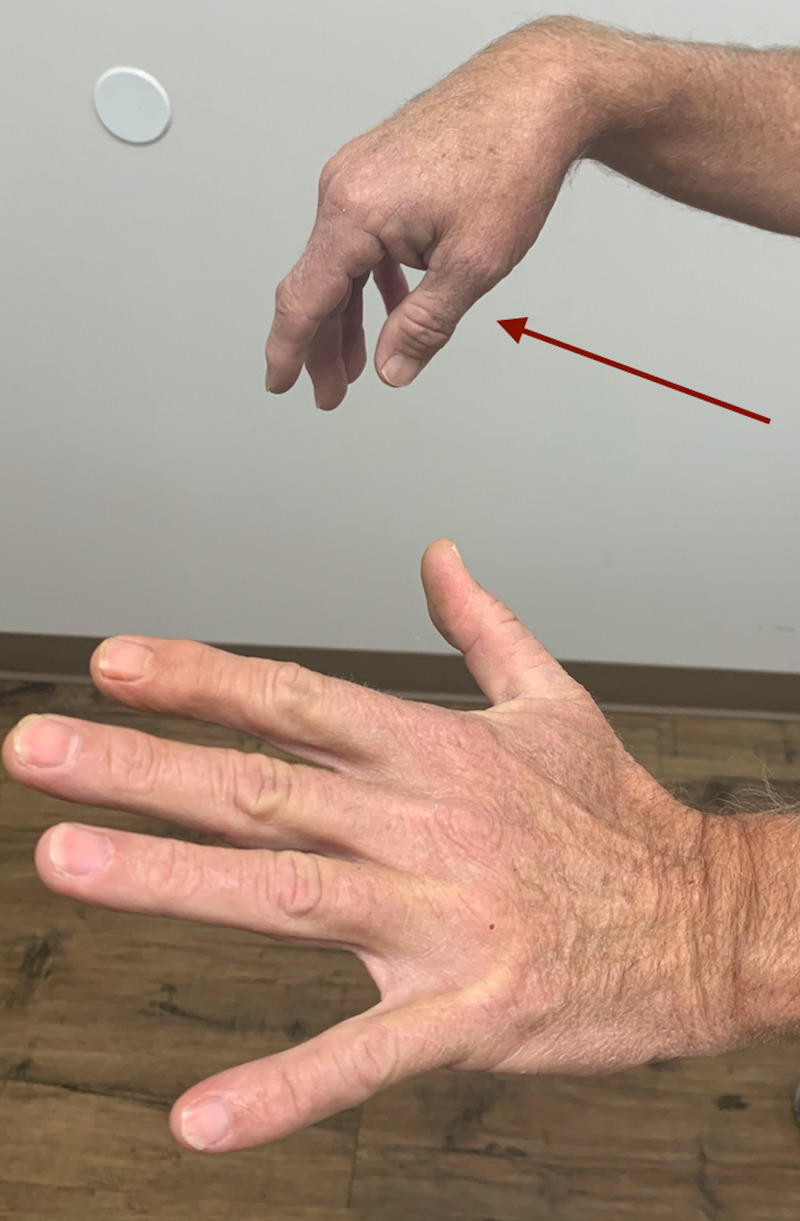
Right wrist, finger, and thumb drop (red arrow)

On examination, we will list the relevant findings. His gait, speech, and cranial nerve examination was entirely normal. Motor examination of the left arm revealed no weakness. A right wrist, thumb, and finger drop were conspicuous (Figure 1).

Right arm power testing is shown below, with wrist, thumb, and finger weakness (Table 1).

**Table 1:** Power testing of the right arm using medical research council (MRC) grading

MUSCLE	POWER GRADE RIGHT ARM
Deltoids	5
Biceps	5
Triceps	5
Brachioradialis	4
Wrist extension	1
Wrist flexion	5
Thumb extension	1
Finger extension	1
Finger flexion	5
Thumb flexion	5
Supinator	3
Pronator teres	5
Finger spreaders (palm on a flat surface)	5

It should be noted that the brachioradialis is relatively spared. This can be explained by the fascicular compartmentalization of the posterior interosseus nerve within the radial nerve proper. Deep tendon reflexes of the upper and lower extremities were entirely normal and symmetric. Sensory examination to pinprick and touch was normal in the hands, including the distribution of the right superficial radial sensory nerve.

A provisional diagnosis of a right radial nerve palsy at the level of the spiral groove was made, based upon the history and pattern of weakness, noting preservation of the power of the triceps muscle and normal triceps jerk.

**Figure 2:**
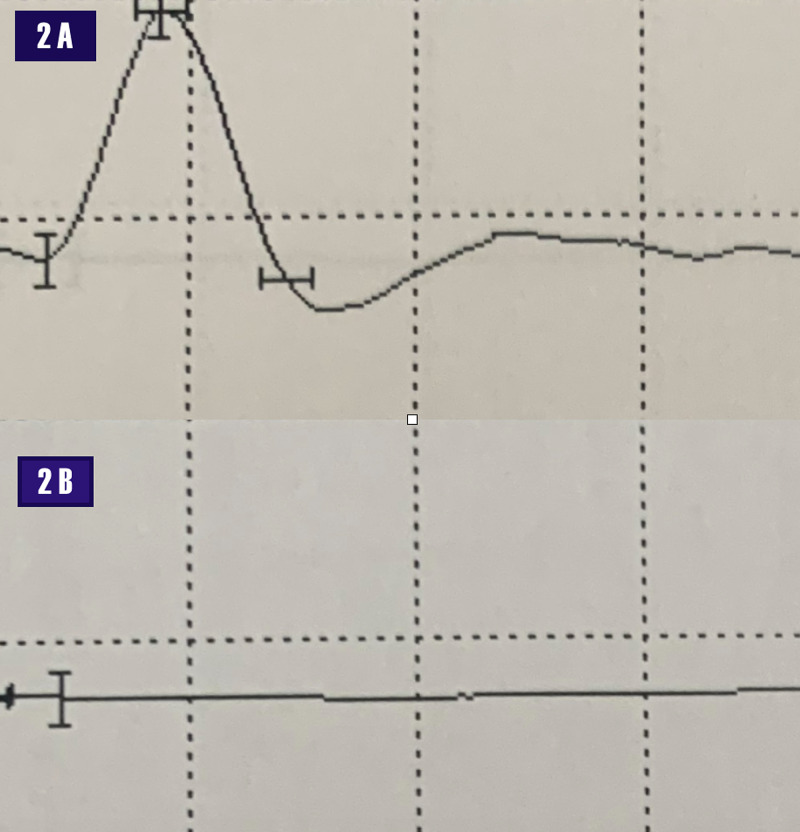
Nerve conduction study. 2A - normal right superficial radial sensory amplitude. Scale: 1 msec along the abscissa and 20 microVolt along the ordinate axis. 2B - absent right radial motor amplitude recording from the extensor indices proprius. Scale: 5 msec along the abscissa and 5 milliVolt on the ordinate axis. Millisecond (msec)

A nerve conduction study revealed normal median and ulnar motor and sensory amplitudes. The radial motor compound muscle action potential (CMAP), recording from the extensor indices proprius (EIP), was absent. The superficial radial sensory amplitude was preserved (Figure 2). 

**Figure 3:**
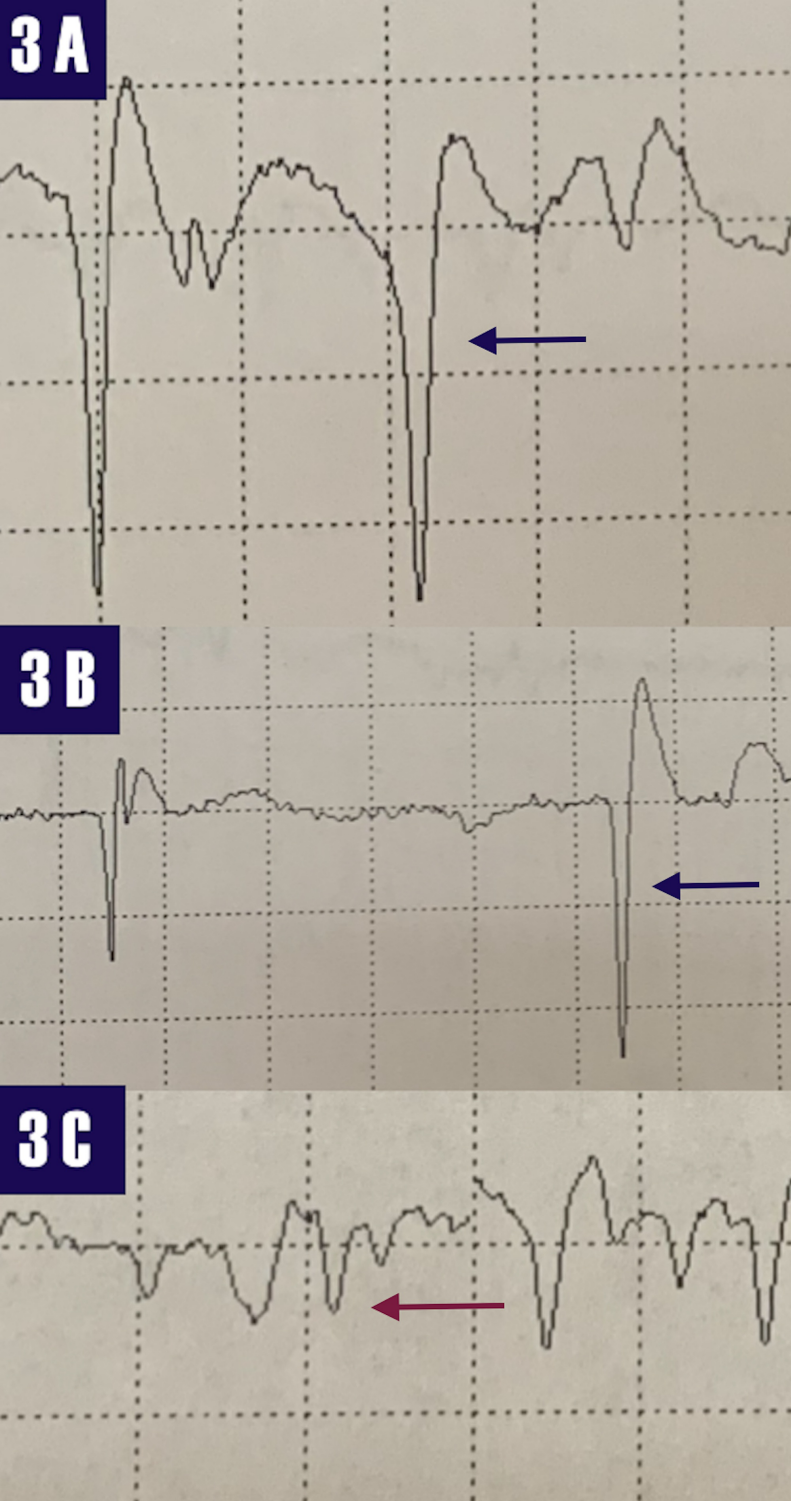
Electromyogram 3A- fibrillation potentials (blue arrow) recording from the right extensor carpi radialis longus. 3B - fibrillation potential (blue arrow) recording from the right extensor digitorum communis. 3C- positive waves (red arrow) recording from the right brachioradialis.

Due to volume conduction, it was not possible to establish a conduction block across the radial motor nerve at the spiral groove of the humerus. An electromyogram revealed florid denervation, fibrillation potentials, and positive waves of the extensor carpi radialis longus ECRL), extensor digitorum communis (EDC), supinator, brachioradialis, and EIP with rapidly firing motor units. The triceps muscle and the cervical paraspinal, deltoids, supraspinatus, pronator teres, brachialis, sublimis, flexor digitorum profundus, and first dorsal interosseus were normal (Figure 3).

The history, pattern of weakness, and nerve conduction study/electromyographic findings are diagnostic of a right radial nerve palsy at the spiral groove. The diagnosis is typically obtained from the history and examination, as the findings are so typical, a "spot diagnosis." It should be noted that the usual mechanism is compression of the radial nerve against the humerus with a focal conduction block. The functional denervation leads to muscle membrane excitability, positive sharp waves, and fibrillation potentials despite axonal continuity. The rapidly firing motor units also imply axonal continuity.

The patient was reassured about the excellent prognosis of this condition, encouraged to attend physical therapy and avoid pressure on the upper arm at the site of the lesion.

## Discussion

The phenotype of wrist, thumb, and finger (WTF) drop has a fascinating history. Pedanius Dioscorides, a physician during the era of Emperor Nero, considered as the father of pharmacology, remarked that "lead makes the mind give way." His magnum opus was the " De Materia Medica," a five-volume encyclopedia on pharmacognosy, a catalog of the pharmacopeia of botanicals. In volume V, he describes wrist drop attributable to lead poisoning [6]. Benjamin Franklin referred to this pattern of weakness as the "dangles" [7]. 

In 1839, Louis Tanquerel des Planches coined the term "encephalopathy" in his treatise "Traite de Maladies de Plomb ou Saturnine," translated from French as "The Treatment of Illnesses arising from Lead or Saturnism" [8].

The word plumbing is derived from the Latin word for lead, " plumbum," in reference to the lead-lined aqueducts that carried water in ancient Roman cities, which led to 100 times the concentration of lead in water compared to modern times. The Romans carried their wine in lead barrels and sweetened it with lead acetate. Remarkably, the Romans were not acquainted with sugar. The hypothesis is that the ruling societies of Rome (the Patricians) developed lead-induced toxic encephalopathy secondary to the excessive drinking of lead-laden wine. The contentious hypothesis is that this led to irrational decision- making that triggered the decline and fall of the Roman empire. However, the fact that lead causes wrist, thumb, and finger drop was a well-established observation [6,9].

The Romans indulged in festivals of excess, revelry, and debauchery while honoring their panoply of deities. These included Bacchus (Dionysus to the Greeks) and Saturn (Cronus to the Greeks). The Roman poet Catullus described it as "the best of times." It was a time to celebrate the winter solstice and the winter planting (sowing) season that lasted seven days; the holiday that morphed into the Christmas holidays, in which Emperor Constantine (the first Christian Roman emperor) began the tradition of gifting. During this time, the citizens of Rome became acutely intoxicated with wine-laden lead acetate and lead ions leaching out of lead-lined wine barrels [9]. The triad of abdominal colic, encephalopathy, and upper limb weakness was associated with the Saturnian festival, which morphed into Saturday night palsy in nineteenth-century England. Spinner et al. adumbrated on this syncretic transformation as a morphing of terminology [10]. Saturday night palsy referred to the weekend revelry associated with the slumber of alcohol over-indulgence and perching the upper arm against a sharp edge with subsequent development of neuropraxia and conduction block [11].

Most toxic polyneuropathies are axonal sensory-motor and length-dependent polyneuropathies with onset in the distal lower limbs. However, acute/sub-acute lead polyneuropathy and the acute porphyric polyneuropathies are unusual; predominantly pure motor, asymmetric, involving the arms initially with weakness of wrist extensors, finger, and thumb-extensors, progressing to proximal arm muscles and ankle dorsiflexors in the later stages. With chronic lead exposure, the polyneuropathy tends to be axonal, sensory-motor, and length-dependent with mild autonomic features [12].

**Figure 4:**
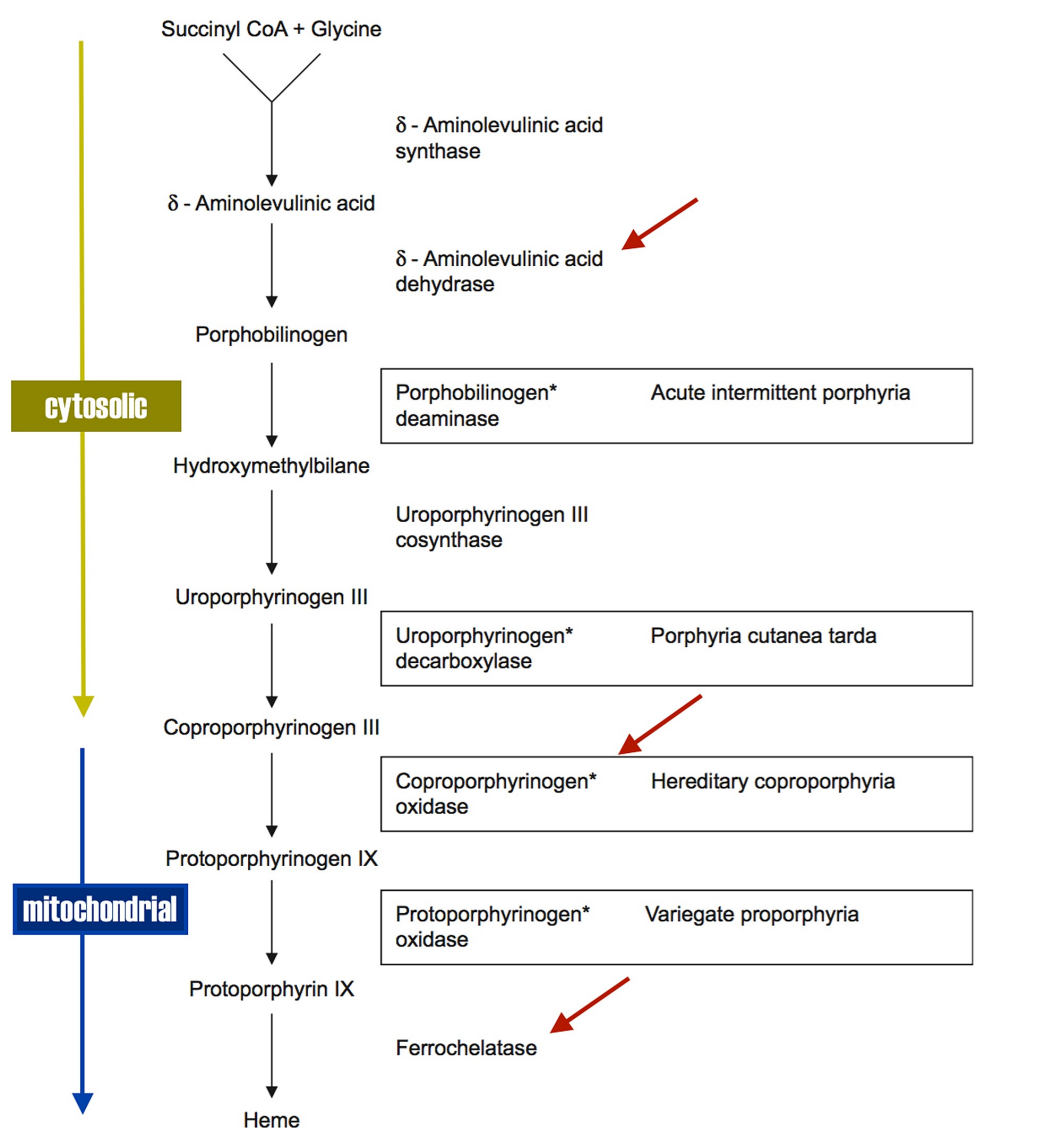
Heme biosynthetic pathway Enzymes inhibited by lead (red arrows) CoA: Coenzyme A The asterisks (*) refer to the four enzyme deficiencies of the acute porphyrias.

Heme is a precursor to hemoglobin, myoglobin, and cytochromes. It consists of four porphyrin rings surrounding a metallic iron atom. Heme is synthesized in the liver and bone marrow and is a conserved process in biology from bacteria (vitamin B12 synthesis) to vertebrates (hemoglobin synthesis). Inborn errors of heme synthesis are referred to as porphyrias. There are four types of acute hepatic porphyrias; acute intermittent porphyria (AIP) accounting for 80 % of all cases. One of the porphyrias, delta-aminolevulinic acid dehydrase (DALA) deficiency, is known as plumboporphyria due to its clinical similarity to lead toxicity. In fact, all the acute porphyrias can potentially mimic the clinical features and motor polyneuropathy of lead toxicity. The polyneuropathy may acutely mimic the Guillain-Barre syndrome (GBS) but without the sensory manifestations. They are listed below as defects in the heme biosynthetic pathway, where the first five steps are cytosolic and the last three steps are mitochondrial (Figure 4) [13-14].

The biochemical profile can be predicted for each genetic defect from the metabolic pathway in Figure 2. The sine qua non of all the acute porphyrias is an elevated serum DALA. The clinical spectrum and motor polyneuropathy of plumbism overlap with that of the acute porphyrias, with the classic triad of abdominal pain (colic), neuropsychiatric manifestations, and a predominantly motor polyneuropathy (Table 2) [12,14].

**Table 2:** The similarities between plumbism and the acute porphyrias (plumboporphyria)

	PLUMBISM	ACUTE PORPHYRIA
Abdominal pain	Yes	Yes
Encephalopathy	Yes	Yes
Hematology	Anemia/basophilic stippling/sideroblastic anemia	Anemia/sideroblastic anemia
Erythrocyte protoporphyrin IX	Yes	Not all the porphyria
Urinary delta-aminolevulinic acid/coproporphyrin	Yes	Yes
Urinary porphobilinogen	No	Yes - Initial screening test
Wrist drop/finger drop	Yes	Yes
Motor neuropathy	Arms first, then legs, asymmetric. The presentation can be acute, sub-acute, or chronic. The chronic form is usually an indolent axonal sensory-motor polyneuropathy.	Arms first, then legs, asymmetric. Usually acute presentation.
Autonomic symptoms	Not common	Yes; usually sympathetic overactivity. But can have urogenital dysfunction and gastrointestinal symptoms.

The overlap even holds at the molecular level where the valence of atomic lead (two) is identical to that of atomic calcium, with lead interfering with the biochemical synthetic pathways of heme. The mechanisms leading to impaired axonal transport and subsequent axonopathy are listed below (Table 3).

**Table 3:** Neurotoxic mechanisms of the acute porphyrias and elemental lead emphasizing similarities Delta-aminolevulinic acid (DALA), gamma-aminobutyric acid (GABA), voltage-gated calcium channels (VGCC), sodium/potassium adenosine triphosphatase (Na/K-ATPase)

	ACUTE PORPHYRIA		LEAD TOXICITY
Windebank et al. [15]	DALA interferes with GABA	Lurban MM [16]	Inhibits DALA dehydratase, coproporphyrin oxidase, and ferrochelatase
Moore M et al. [17]	DALA-induced neurotoxicity via free radical formation	Suszkiw JB [18]	Lead interferes with pre-synaptic VGCC and inhibiting the synaptic release of neurotransmitters
Sengupta A et al. [19]	Inadequate heme synthesis - dysfunction of Na/K ATPase and axonal transport impairment	Krishnan AV et al. [20]	Impaired Na/K-ATPase pump - axonal depolarization - and membrane hyperpolarization and refractoriness

## Conclusions

Wrist, thumb, and finger drop due to neuropraxia at the spiral groove of the humerus is quite common, as are the other etiologies of wrist drop, traumatic and lesional. The clinical presentation in many instances can be made on the spot, the so-called "spot diagnosis." In medical historiography, this clinical phenotype has a spectacular history with ebbs and flows that reflected technological advances. This article was written in order to resuscitate this rich and fascinating history. The clinical parallelism between plumbism and the porphyrias is reflected at the molecular biology level. The lessons learned from history need to be heeded as epidemics of lead poisoning continue to happen.
